# Effectiveness of a Simplified Checklist to Overcome the Inertia of Treatment Implementation in ACS Patients with High Comorbidity Burden

**DOI:** 10.3390/jcm14072469

**Published:** 2025-04-04

**Authors:** Jonathan X. Fang, Yap-Hang Chan, Zaid I. Almarzooq, Cheung-Chi Simon Lam, Yiu-Tung Anthony Wong, Han Naung Tun, Kai-Hang Yiu, Hung-Fat Tse, Hon-Wah Chan, Chor-Cheung Frankie Tam

**Affiliations:** 1 Cardiology Division, Queen Mary Hospital, University of Hong Kong, Hong Kong SAR, China; 2 Department of Cardiology, National Heart Centre Singapore, Singapore 169609, Singapore; 3 Veterans Affairs Boston Healthcare, West Roxbury, MA 02132, USA; 4 Hong Kong Sanatorium and Hospital, Hong Kong SAR, China; 5 Larner College of Medicine, University of Vermont, Burlington, VT 05405, USA

**Keywords:** acute coronary syndrome, implementation research, checklist, clinical care pathways, treatment inertia, high comorbidity burden, Charlson Comorbidity Index

## Abstract

**Background/Objective**: High-risk subsets of patients with acute coronary syndrome (ACS) experience decreased access to optimal care and have poor clinical outcomes, reflecting an inertia to the delivery of guideline-directed and evidence-based therapy and implementation of critical care pathways. We aim to investigate the clinical effectiveness of a simplified implementation checklist to counter treatment inertia in patients with high comorbidity burden. **Methods**: An ACS critical care pathway was simplified and reduced to a minimalistic checklist including only items on GDMT and invasive strategy. A total of 2005 consecutive patients with ACS were evaluated including 1499 patients receiving standard care and 506 patients managed with the checklist. Patients with STEMI undergoing primary percutaneous coronary interventions and patients receiving upfront cardiovascular intensive care were excluded. Multivariate regression spline models were used to study the relationship between comorbidity, expressed as the Charlson Comorbidity Index (CCI) and a management strategy including guideline-directed medical therapy (GDMT) and an early invasive approach. Inverse probability of treatment weighting (IPTW) was used to address confounding factors. The use of GDMT and early invasive therapy were compared in patients receiving standard care and checklists. The 90-day composite outcome of all-cause mortality, recurrent ACS and stroke were compared between patients receiving standard care and those receiving checklists. **Results**: High CCI was associated with decreased GDMT, invasive strategy and the utilization of critical care pathway. Checklist utilization was unaffected by high CCI and led to sustained and higher use of GDMT and invasive approach in patients despite high CCI. Checklist managed patients have >10% higher rates of prescription of each class of GDMT (*p* < 0.0001) and more than twice the rate of early invasive approach (51.0% vs. 20.7%, (*p* < 0.0001) compared to patients receiving standard care. The 90-day composite outcome was lower in checklist management patients compared to patients receiving standard care, adjusted hazard ratio 0.61 (95% CI 0.46–0.81), log-rank *p* = 0.0006, especially in patients with high CCI, adjusted hazard ratio 0.60 (95% CI 0.38–0.97), log-rank *p* = 0.035 for CCI 5–6; adjusted hazard ratio 0.53 (95% CI 0.35–0.84), log-rank *p* = 0.0057 for CCI 7 or more. **Conclusions**: The use of a simplified checklist is associated with better implementation of GDMT and invasive strategy as well as better 90-day clinical outcomes in ACS patients with high comorbidity burden

## 1. Introduction

The management of Acute coronary syndrome (ACS) has improved significantly in the last decade [1] with the improvement in medical therapy such as the introduction potent P2y12 inhibitor and PCSK9 inhibitors for potent lipid lowering, as well as improved revascularization technologies and techniques, such as the increased use of intravascular imaging and the adoption of the transradial access first approach and improved coronary stents for percutaneous coronary intervention (PCI) [2,3]. To promote the implementation of improvement treatments, quality matrices and critical care pathways [4,5,6,7] have been developed. However, treatment inertia is a persistent problem affecting the management of high-risk patient subsets such as elderly patients and those with high comorbidity burden [8,9].

Treatment inertia can be due to patient factors and physician factors. Patient factors include polypharmacy, high bleeding risk, active organ dysfunction and uncertain overall prognosis, and geriatric syndromes such as dementia and frailty [10]. Physician-related factors are less well defined and may include ineffective cross-specialty collaboration and risk estimate aversion of non-specialists [11]. A previous study has shown the decreased utilization of timely cardiac catheterization and therapeutics when ACS patients are admitted under units where the managing physicians are of a different specialty, such as general internal medicine or hospital medicine [12]. Concomitant diseases requiring active treatments often lead to the admission of ACS patients with multimorbidity to medical intensive care units, high-dependency units and general internal medicine wards rather than coronary care units (CCUs) and cardiovascular intensive care units (CICUs). The overall management strategy of these patients is not straightforward and can rarely be dictated by the critical-pathway guided algorithmic care offered by modern specialized cardiovascular care units in most institutions. We aim to study the applicability of minimizing critical care pathways into a checklist to increase the implementation of guideline-directed medical therapy and timely cardiac catheterization and revascularization for ACS patients who do not receive upfront CCU and CICU care.

## 2. Methods

### 2.1. Study Population

**Figure 1:**
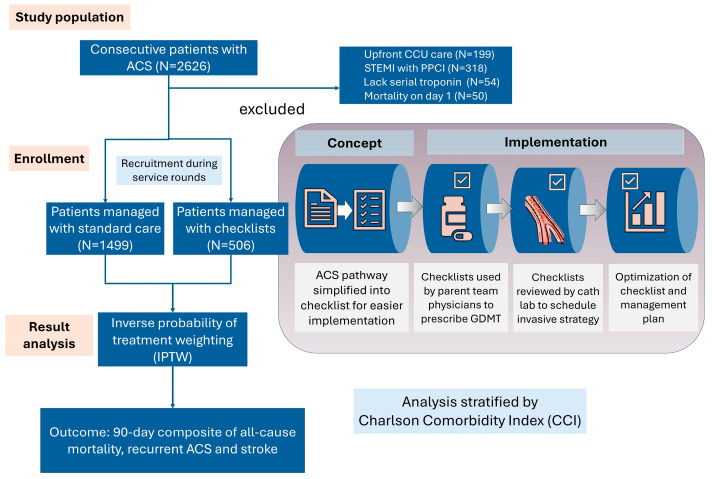
Study design. Critical care pathways were minimalized and reduced to checklists containing items for intended guideline-directed medical therapy (GDMT) and risk stratification revascularization strategy only. Consecutive patients with ACS were included. Inverse probability of treatment weighting (IPTW) was used to balance covariates in standard care and checklist-managed patients. The clinical outcome was the 90-day composite of all-cause mortality, recurrent acute coronary syndrome (ACS) and stroke. Survival analysis was stratified according to tertiles of Charlson Comorbidity index (CCI). CCU: coronary care unit. STEMI: ST-elevation myocardial infarction. PPCI: primary percutaneous coronary intervention.

Consecutive patients admitted with ACS to the medical department of a teaching hospital in Hong Kong (Queen Mary Hospital, University of Hong Kong, Hong Kong SAR, China) from January 2016 to June 2019 were evaluated retrospectively (N = 2626). The hospital has a case volume of approximately 800 PCI per year including 150 primary PCI, two active and one back up cardiac catheterization laboratory, and 24-h emergency admission and primary percutaneous coronary intervention (PPCI) service. Patients with ST-elevation myocardial infarction (STEMI) who underwent primary PPCI (N = 318), patients treated with critical care pathways in the coronary care unit (N = 199) and patients lacking serial troponin measurements (N = 54) were also excluded (Figure 1). Patients were recruited during service rounds twice daily. We excluded patients who died on the first day of hospitalization (N = 5) to mitigate the possibility of selection bias arising from patients who died upon admission before service rounds in the standard care group. A total of 2005 patients were included in the analysis, including 506 patients receiving checklist care and 1499 patients receiving standard care.

Under standard care, patients with a high comorbidity burden who did not receive upfront coronary care service or admission to a cardiology unit would be managed in non-cardiology units, such as general internal medicine units. The primary management team provided the initial management plan as well as twice-daily service rounds. Cardiologists provided initial consultation assessment upon the request of the parent team, followed by daily consultation rounds if necessary. Cardiology consultation was not mandatory. Interventional cardiology service would be notified by the clinical cardiology consultation service if the patient required cardiac catheterization.

Under checklist care, the interventional cardiology service would automatically be notified of the patient presentation to stratify for coronary workup and revascularization. All checklists were reviewed by the interventional cardiology in the morning for stratification and scheduling of cardiac catheterization. Medications would also be reviewed by the cardiology service and suggestions for further optimization could be made without the need for the parent team to formally initiate a cardiology consultation request. The decision to initiate care with the checklist were made during services rounds based on clinical decisions by the treating physicians.

### 2.2. Design and Implementation of the Checklist

The decision to design a simplified clinical checklist was made by the interventional cardiology service of the hospital. The aim was to provide a streamlined one-step implementation tool to standardize the management of patients with ACS who received care by non-cardiologists and provided invasive management early to patients in need. Although the use of the checklist was encouraged, it was not mandatory. It was hypothesized that simplicity was the key to increasing implementation rate in the presence of multimorbidity. Hence, the critical care pathway for ACS was reduced to a single-page implementation checklist without algorithms. The checklist only required the treating physicians to select the intended medical therapy and decision to consult cardiology. Medications included 5 classes of guideline-directed medical therapy (GDMT) for ACS, including aspirin, P2y_12_ inhibitors, statin, beta-blocker, and drugs with antagonistic effects on the angiotensin system, including angiotensin converting enzyme inhibitor (ACEI), angiotensin receptor blocker (ARB) or angiotensin receptor blocker-neprilysin inhibitor (ARNI).

**Figure 2:**
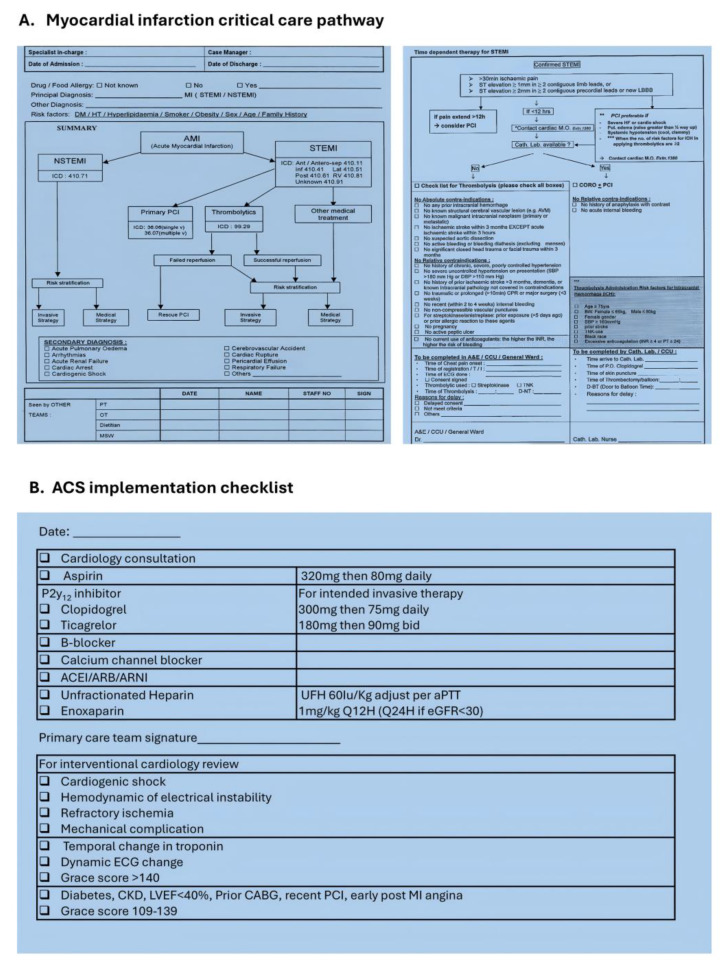
Difference between comprehensive critical care pathway aim specifically for managing patients with ACS (**A**) and simplified ACS implementation checklist for reinforcing care of ACS for patients with concomitant diseases (**B**). While traditional critical care pathways include management algorithms and management subsections with multiple items, the checklist is a minimalistic intervention tool that requires the managing physician to only indicate the intended medical therapy, foregoing all complexities and algorithmic care which might not be applicable to patients with high comorbidity burden and concomitant active illness requiring in-hospital treatment. The checklists and electronic health records are then reviewed by the interventional cardiologist and staff of the cardiac catheterization laboratory, allowing the simplified stratification and scheduling of cardiac catheterization for patients who may potentially benefit from revascularization.

A simplified risk assessment tool was included on the checklist to indicate the urgency of needing cardiac catheterization. Items included were cardiogenic shock, hemodynamic or electrical instability, refractory ischemia and mechanical complication, which were the highest priority; a temporary change in cardiac troponin, a dynamic change on the electrocardiogram and a Global Registry of Acute Cardiac Event (GRACE) score of more than 140 [13] were of a high priority, and the presence of comorbidities such as diabetes prior to revascularization and a left ventricular ejection fraction of less than 40% or a GRACE score of 100–139 were of an intermediate priority. Risk stratification was centralized by having all checklists reviewed by interventional cardiologists daily (Figure 2). The checklists were available for use by parent team physicians across internal medicine units, intensive care and high dependency medical units, and specialty units without the need for additional cardiology consultation requests, as well as in surgical units upon consultation of the cardiology service.

### 2.3. Data Source and Definitions

Data collection was performed retrospectively. Information on patient demographics, comorbidities, laboratory results, in-hospital and discharge medications, and cardiac catheterization information, were retrieved from the territory-wide electronic patient records database known as the Clinical Data Analysis and Reporting System (CDARS), which has very high data accuracy and low percentages of missing information and covers over 90% of territory-wide patient care information across more than 40 institutions. Elderly was defined as the age of 75 or greater based on an existing definition [14]. The early invasive approach was defined as cardiac catheterization within the first 72 h of hospitalization. The Killip classification [15] was used for risk stratification to align with the practice pattern of internists, with class I defined by clear chest auscultation finding, class II defined by audible crepitations on physical examination without pulmonary edema on chest X-ray, class III defined by visible pulmonary edema present on chest X-ray, and class IV define by hypotension. The Charlson comorbidity index (CCI) [16] was calculated and divided into patient tertiles, with tertile 1 being lowest CCI and tertile 3 being the highest. Ninety-day clinical outcome information on recurrent ACS and stroke was obtained from all subsequent planned or unplanned patient encounters including hospitalization and emergency department and clinic visits across 43 hospitals and clinics in the territory. Ninety-day all-cause mortality data were retrieved from the regional death registry.

### 2.4. Statistical Analysis and Clinical Endpoints

Logistics regression and linear regression were used to create regression models with restricted cubic spline to visualize the relationship between CCI and the likelihood of receiving checklist management or critical pathway management, the likelihood of receiving early invasive therapy, as well as the total number of classes of GDMT received. Ten splines were used for CCI. The results were plotted using the Lowess smoothing function.

A multivariate logistic regression model was used to obtain propensity scores for the likelihood to be managed with the checklist. The model included 35 variables, including five multi-categorical variables, and with the independent variable set as checklist management. The model was assessed with a Hosmer-Lemeshow test for goodness of fit by Pseudo R^2^ and C-statistic for discrimination (Supplementary Materials). The inverse probability of treatment weighting (IPTW) was used for generating pseudo-populations with counterfactual inference to balance the covariates between patients receiving standard care and checklist management. The standardized mean difference (SMD) was used to assess the balance of covariates between the two groups. A well-balanced covariate was defined as having an SMD of <0.2. A kernel density plot was used to visualize the overlap of propensity score in the checklist and non-checklist population before and after IPTW (Supplementary Materials). Continuous variables in the IPTW-generated pseudo-population were expressed as linearized estimated means and compared using the two-sample *t*-test, while dichotomous variables were expressed as frequencies and percentages and compared using the chi-square test. Continuous variables in the baseline population were also expressed as means and standard deviation to allow a comparison with the IPTW linearized estimated means.

The clinical endpoint was defined as the 90-day composite of all-cause mortality, recurrent ACS and stroke. A comparison was conducted for patients receiving checklist care and those receiving standard care after IPTW, and visualized using weighted Kaplan–Meier estimates. A weighted log-rank test was used to determine the statistical significance of time-to-event results, while weighted Cox proportional hazard models were used to obtain the adjusted hazard ratio and 95% CIs. The analysis was stratified by CCI tertiles. Tests for proportional hazard assumption and the visualization of Schoenfeld residue were used to test the validity of the Cox proportional hazard models.

The effect modification of the checklist on 90-day outcomes was assessed for patient subgroups including CCI tertiles, comorbidities, ACS presentation, ward assignment and treatment assignment. Multiple sensitivity analyses were performed, including a Cox proportional hazard model including all variables, the inclusion of covariates with SMD ≥ 0.1 after IPTW in multiple cox models, a 1:1 propensity score matching using a caliper of 0.2 of the square root of the standard deviation, the and remodeling of CCI as a continuous variable. A two-sided *p*-value < 0.05 was considered statistically significant. Data analysis was performed using Stata SE 18 (StataCorps, College Station, TX, USA).

### 2.5. Ethics

The Institutional Review Board of the Hong Kong Hospital Authority West cluster and University of Hong Kong has approved the study. The study was carried out in accordance with good clinical practice guidelines laid out in the declaration Helsinki.

## 3. Results

### 3.1. Baseline Characteristics

The baseline population included a total of 2005 patients with high comorbidity burden and more advanced age than the usual ACS population. A total of 1499 patients received standard care and 506 patients were managed with the checklist. The mean age was 75 years. Comorbidity burden was high, with a mean CCI of 5.5, where a CCI of 5 or more was considered high according to the literature [17]. A total of 11.1% of patients had underlying malignancy, including 9% with systemic or metastatic disease. More than half of the patients had chronic kidney disease stage 3 or above. A total of 24.7% of patients had ACS as a secondary diagnosis and another active disease as the main reason for hospitalization. A total of 13.9% of patients had delayed presentation of STEMI, 8% of patients had prior gastrointestinal bleeding, 11.3% had atrial fibrillation and 9% had dementia. All covariates were well balanced, with SMD < 0.2 after IPTW. Baseline characteristics are listed in Table 1.

### 3.2. Implementation Rates by CCI in Standard and Checklist Care

**Figure 3:**
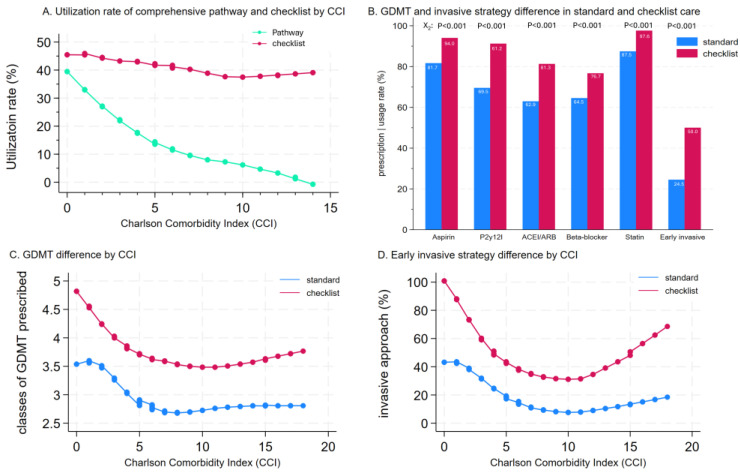
Relationship between CCI and treatment implementation. (**A**). Utilization rate of checklist and traditional critical care pathway with increasing CCI shows decreased utilization rate of pathway with increasing CCI and relatively constant adoption rate of checklist irrespective of CCI. (**B**) In the baseline population, GDMT and early invasive therapy are significantly higher in patients managed with the checklist compared to standard care. (**C**,**D**) The GDMT prescription rate and invasive therapy implementation rates are significantly higher in checklist-managed patients compared to patients receiving standard care. Moreover, the implementation rates increase towards, with CCI beyond 10 in the checklist group, whereas implementation rates drop and plateau with CCI > 5 with standard care.

Regression spline models showed that the utilization rate of traditional critical care pathway drops as CCI increases, whereas the utilization rate of the simplified checklist is maintained over all CCI rates (Figure 3A). Patients managed with the checklist showed a significant increase in each class of medications prescribed compared to those in standard therapy with prescription rates that were at least 10% higher in the checklist group for each class of GDMT (all *p* < 0.001 as well as the doubling of the rate of early invasive approach *p* < 0.001). The result was consistent before (Figure 3B) and after IPTW (Table 2). With GDMT and the early invasive approach, the implementation rate decreases and then plateaus, with CCI increasing beyond 5 with standard care alone, while the implementation rate is maintained and follows U-shape relationships with increased utilization towards a higher CCI beyond 10 in checklist-managed patients (Figure 3C,D).

In addition to a significantly higher overall GDMT prescription rate, there were also higher rates of use of potent p2y_12_ inhibitor ticagrelor (19.4% vs. 11.0%, *p* = 0.003) and an increased prescription rate of moderate intensity statin (69.5% vs. 52.3%, *p* < 0.0001) in the checklist group compared to the standard care group. Heparin was used in 58.9% of patients in standard therapy and 85.0% of patients in checklist therapy (*p* < 0.0001)

### 3.3. Ninety-Day Clinical Outcomes

**Figure 4:**
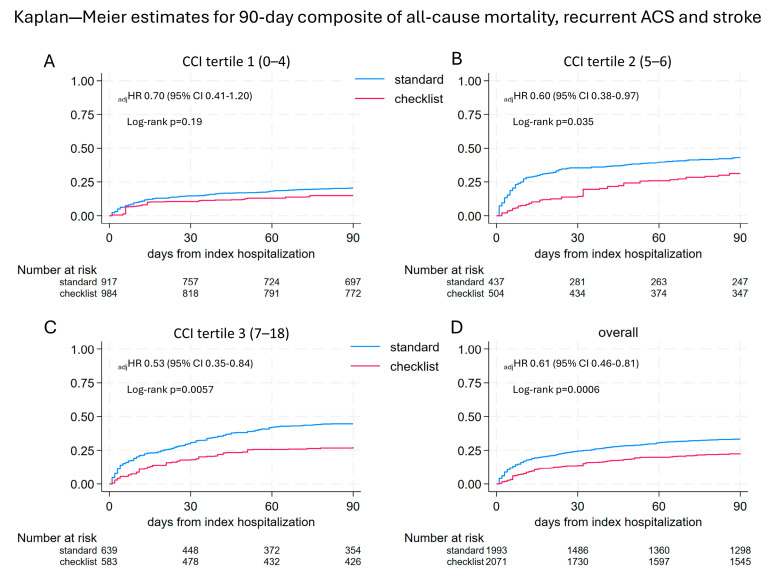
Kaplan–Meier estimates of 90-day composite of all-cause mortality, recurrent ACS and stroke for checklist and standard-care patients, according to CCI tertiles and in overall cohort. With an increasing CCI tertile, use of the checklist was associated with more evident difference in 90-day event rates. For the CCI tertile 1 (0–4), the result did not reach statistical significance for the given sample size, adjusted hazard ratio 0.7 (95 0.41–1.2), log-rank *p* = 0.19. For CCI tertile 2 (5–6), there were significantly fewer events with checklist care, adjusted hazard ratio 0.60 (95% CI 0.38–0.97), log-rank *p* = 0.035. For CCI tertile 3 (7–18), there was a further increase in difference between checklist and standard care, adjusted hazard ratio 0.53 (95% CI 0.35–0.84), *p* = 0.0057 (**A**–**C**). In the overall cohort, use of the checklist was also associated with fewer events, adjusted hazard ratio 0.61 (95% CI 0.46–0.81, log-rank *p* = 0.0006 (**D**).

Loss-to-follow-up occurred in 3.7% of checklist-managed patients and 2% of patients receiving standard care, which was comparable (chi-square *p* = 0.27). Estimated 90-day all-cause mortality data retrieved from territory-wide death registry, including all reports in and outside of healthcare setting unaffected by loss-to follow-up, were 14.6% in checklist-managed patients and 25.1% in the standard care patients (chi-square *p* = 0.0006). Survival analysis showed that, with increasing CCI, checklist care was associated with a progressively lower event rate compared to standard care (Figure 3). For the CCI tertile 1 (CCI 0–4), event rates were low in both the checklist group and standard care, with a hazard ratio 0.70 (95% CI 0.41–1.20) which did not reach statistical significance. For the CCI tertile 2 (CCI 5–6), there was a significantly lower event rate with the checklist vs. standard care, the adjusted hazard ratio was 0.60 (95% CI 0.38–0.97) and log-rank *p* = 0.035. For the CCI tertile 3 (CCI 7 or more), the split between the checklist and standard care further increases, hazard ratio 0.53 (95% CI 0.35–0.84), log-rank *p* = 0.0057. Overall, checklist care was associated with fewer 90-day composite events and a hazard ratio of 0.61 (95% CI 0.46–0.81, *p* = 0.035) (Figure 4).

### 3.4. Subgroup and Sensitivity Analysis

**Figure 5:**
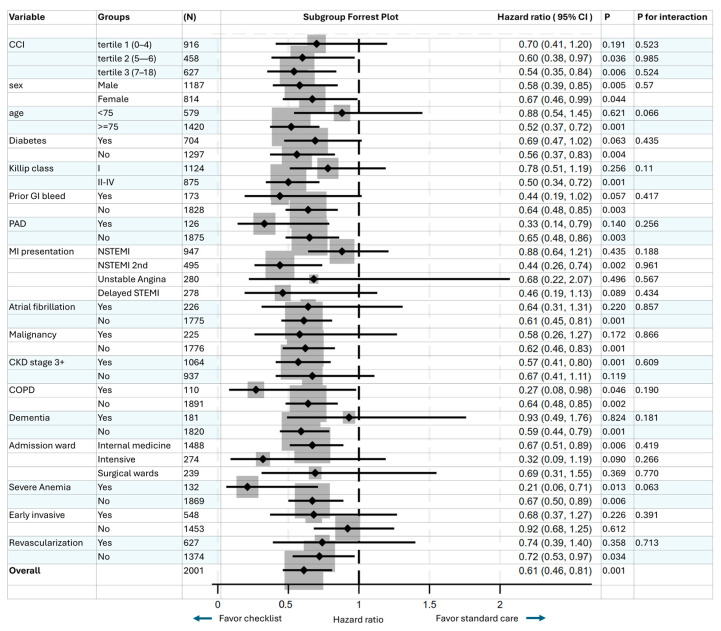
Subgroup analysis and test for effect modification. There was overall no effect modification found on the effect of checklist care on 90-day outcomes for subgroups according to CCI, sex, elderly age, diabetes, Killip class (I vs. II—IV), prior GI bleeding, peripheral vascular disease, myocardial infarction (MI) presentation, atrial fibrillation, malignancy, chronic kidney disease stage 3 or above, chronic obstructive pulmonary disease (COPD), dementia, admission ward, use of early invasive therapy, and all subsequent revascularization (*p* for interactions all > 0.05). All subgroups had hazard ratios < 1.

Overall, no effect modification was found for the checklist and 90-day outcomes across multiple subgroups (all *p* for interactions > 0.05), with overall consistent results with checklist use having hazard ratios < 1. Hazard ratios were closer to one (>0.75) in patients without an early invasive strategy, patients with dementia, age < 75 years, or Killip class I, suggesting a more neutral effect, while the hazard ratios were lower (<0.5), suggesting more pronounced effects in patients with severe anemia, NSTEMI as secondary diagnosis, delayed presentation of STEMI, chronic obstructive pulmonary disease (COPD), admission under medical intensive care and peripheral artery disease. The hazard ratios were lower with increasing CCI tertiles. These findings were only exploratory as the study was not powered to show statistical significance for the subgroup analysis, with some of the 95% CIs crossing 1 (Figure 5).

Multiple sensitivity analysis converged on similar results showing hazard ratios favorable checklist management. IPTW followed by multivariate Cox regression by including covariates with SMD ≥ 0.10 in a multivariate Cox proportional hazard model, including the covariate for heart failure (SMD 0.10), unstable angina (SMD 0.13), prior ACS (SMD 0.15) and admission to specialty ward (SMD 0.10). The results are shown in Table 3. The assumption of proportional hazard was not violated for the Multivariate Cox proportional hazard model used (Supplementary Table S2, Supplementary Figure S2). The sample size was reduced to 416 patients per group after propensity score matching, resulting in an underpowered analysis.

## 4. Discussion

Critical care pathways for ACS improve short-to-midterm clinical outcomes [5,6,7] but may be difficult to implement outside of the setting of coronary care and cardiovascular intensive care units. Real world data showed an under-utilization of coronary revascularization in elderly patients [18,19] as well as degradation of overall quality of care in high-risk patients with ACS [20]. The Charlson Comorbidity index (CCI) has been shown to correlate with long term clinical outcomes in ACS [17] and is the currently recommended method of assessing comorbidity burden in patients with ACS according to European Society of Cardiology guidelines [21]. Our study shows that a high comorbidity burden according to CCI decreases utilization rate of critical care pathway as well as decreased use of guideline directed medical therapy (GDMT) and cardiac catheterization under standard care. The baseline population in our study has higher mean age, comorbidity burden and complex presentation than most reported cohorts in the literature. One out of four patients in our study had ACS presenting as a concurrent condition and another active disease presenting as the primary reason for hospitalization. We are the first to show that simplicity of intervention is associated with higher implementation rate and better patient outcomes. With a simplified checklist, GDMT and early invasive approach can be maintained in patients with CCI. Ninety-day composite of all-cause mortality and major adverse cardiovascular events were also lower, especially in patients with higher comorbidity burden. Subgroup analysis showed the generalizability of our finding while multiple sensitivity analysis consolidates the robustness of the results. We have excluded patients who received upfront critical pathway care, such as patients with STEMI receiving primary PCI and those admitted to cardiovascular intensive care unit and coronary care units upfront, as these patients were already receiving optimal care under detail critical care pathways. We excluded patients who died on the first day of hospitalization to avoid selection bias favoring the checklist as recruitment to the checklist did not occur immediately during hospitalization. We did not enforce the use of the checklist or employ randomization as the goal of the study was to observe real life challenges in implementation due to comorbidity burden.

While CCI offers a simple method to quantify comorbidity burden, management strategies should be tailored to the needs of individual patients. For example, tailored decisions on the ideal antithrombotic regimen are required in the presence of anemia, prior GI bleeding, malignancy, thrombocytopenia, recent surgery and concomitant anticoagulation. Optimal timing and indication of cardiac catheterization with possible revascularization should be individualized in the presence of acute kidney injury or other organ dysfunction, active sepsis or neurological events, frailty, geriatric syndromes, malignancy with uncertain overall patient prognosis and delayed presentation of STEMI. These clinical decisions are complex and often require a multidisciplinary approach with active collaboration between cardiologists and other specialists to achieve a balanced and wholistic management plan.

ACS patients have been shown to have lower utilization or resources including medical therapy, specialist care and cardiac procedures, as well as higher one-year mortality when there is inadequate specialist input [12]. Ambiguity of management plans and differences on perceived risk-benefit ratios of individual treatments among physicians of different specialties could jeopardize overall patient care. Major physician-related factors of treatment inertia may include unfamiliarity to evidence-based practice outside of one’s expertise, especially in the presence of conflicting evidence, or decreased incentive to pursue aggressive management strategies when perceived treatment risk is high [11]. The subgroup analysis of our study showed trends of benefits particularly in difficult-to-manage patient subsets such as those with ACS as a secondary condition, delayed presentation of STEMI and severe anemia. Simplicity and maintaining flexibility of patient care without conforming to stringent algorithm-based care should be a key consideration in devising implementation tools for patients with high comorbidity burden.

### Limitations and Future Directions

Our study used robust epidemiological methods and multiple sensitivity analysis to address confounding factors. The rate of loss-to-follow-up and missing data was low owing to the availability of territory-wide data across multiple institutions. However, this study has several limitations. The inherent limitation of the retrospective cohort studies allow only association and not causality to be drawn. Despite robust methods of addressing confounders, unmeasured residue confounders may exist, such as physician motivation and patient frailty, smoking status and non-familial hypercholesterolemia. Prior revascularization rate was under-reported in our population. In this predominantly elderly population with high comorbidity burden, the distinction of myocardial infarction according to the Fourth Universal Definition of Myocardial Infarction [22] into type 1 and type 2 myocardial infarction could not be reliably made owing to the complexity of clinical presentation of the patients and the low rate of cardiac catheterization. Our study focuses on clinical outcomes and did not study the outcomes used in implementation science, such as fidelity, sustainability, and appropriateness of the checklist. Further implementations study with hybrid design are required to investigate implementation inertia of ACS and to validate our result in a different population.

## 5. Conclusions

ACS patients with high comorbidity burden have restricted access to medical therapy, coronary revascularization and specialized care. The use of a simplified checklist is associated with a better delivery of medical therapy and coronary revascularization, regardless of comorbidity burden. Use of the checklist is also associated with lower short-to-mid-term rate of mortality and cardiovascular events, especially in patients with high comorbidity burden.

## Figures and Tables

**Table 1:** Baseline characteristics and standardized mean difference (SMD) before and after inverse probability of treatment weighting (IPTW).

	Baseline			IPTW		SMD (Baseline)	SMD (IPTW)
	Overall (N = 2005)	Standard (N = 1499)	Checklist (N = 506)	Standard (N = 1994.48)	Checklist (N = 2071.37)		
Baseline Characteristics	Mean (SD)	Mean (SD)	Mean (SD)	Mean	Mean		
Age (years)	74.53 (14.3)	74.71 (14.55)	74.02 (13.59)	74.56	74.00	−0.05	−0.04
Male sex	59.3%	57.5%	64.6%	59.2%	61.0%	0.15	0.04
Underlying malignancy	11.2%	11.4%	10.7%	11.2%	11.6%	−0.02	0.01
Metastatic cancer	9.0%	9.2%	8.5%	9.1%	9.3%	−0.02	0.01
Diabetes	35.2%	34.1%	38.3%	35.5%	36.0%	0.09	0.01
Diabetic microvascular ds	19.7%	19.7%	19.8%	19.8%	18.1%	0.00	−0.04
Familial hyperlipidemia	0.8%	0.9%	0.8%	0.8%	0.7%	−0.01	−0.01
Dementia	9.0%	10.4%	5.1%	9.0%	6.6%	−0.20	−0.09
Hypertension	53.9%	54.4%	52.4%	54.4%	57.1%	−0.04	0.05
Prior ACS	20.4%	21.9%	16.0%	20.4%	16.1%	−0.15	−0.11
Prior CAD	3.1%	3.1%	3.2%	3.0%	2.0%	0.00	−0.06
Atrial fibrillation	11.3%	11.6%	10.3%	11.3%	11.3%	−0.04	0.00
Heart failure	17.5%	18.6%	14.4%	17.7%	14.0%	−0.11	−0.10
Stroke	16.2%	17.0%	14.0%	16.0%	14.4%	−0.08	−0.04
Peripheral artery disease	6.3%	6.0%	7.3%	6.4%	6.6%	0.05	0.01
Prior GI bleeding	8.6%	9.0%	7.5%	8.7%	8.3%	−0.06	−0.01
CKD stage							
1 or none	15.9%	15.7%	16.6%	15.8%	15.0%	0.02	−0.02
2	30.9%	29.9%	33.8%	30.6%	30.9%	0.08	0.01
3	29.6%	29.3%	30.4%	29.9%	33.7%	0.02	0.08
4	12.1%	13.0%	9.7%	12.1%	10.2%	−0.10	−0.06
5	11.4%	12.1%	9.5%	11.7%	10.2%	−0.08	−0.05
Connective tissue disease	2.0%	1.8%	2.8%	2.0%	2.1%	0.06	0.01
Syncope	0.7%	0.8%	0.6%	0.7%	0.4%	−0.03	−0.04
COPD	5.5%	5.2%	6.5%	5.5%	5.9%	0.06	0.02
Prior PCI	0.9%	0.5%	2.0%	0.7%	0.7%	0.13	0.01
Prior CABG	1.4%	1.5%	1.0%	1.4%	1.0%	−0.05	−0.04
ACS type							
NSTEMI	47.4%	40.7%	67.0%	47.1%	41.1%	0.55	−0.12
NSTEMI as a secondary diagnosis	24.7%	27.5%	16.6%	24.8%	25.7%	−0.26	0.02
Unstable angina	14.0%	15.7%	9.1%	14.1%	18.8%	−0.20	0.13
Delayed presentation of STEMI	13.9%	16.1%	7.3%	14.0%	14.4%	−0.28	0.01
Admission year							
2016	26.4%	32.2%	9.1%	26.5%	27.1%	−0.60	0.01
2017	31.8%	29.2%	39.7%	31.7%	30.7%	0.22	−0.02
2018	28.0%	24.9%	37.0%	28.2%	28.6%	0.26	0.01
2019	13.8%	13.6%	14.2%	13.6%	13.7%	0.02	0.00
Admission unit							
female ward 1	11.3%	11.4%	11.1%	11.2%	11.4%	−0.01	0.01
female ward 2	11.9%	12.0%	11.9%	12.2%	11.2%	0.00	−0.03
female ward 3	10.3%	10.8%	8.9%	10.3%	8.6%	−0.06	−0.06
male ward 1	14.7%	12.8%	20.4%	14.4%	14.7%	0.20	0.01
male ward 2	15.1%	14.0%	18.6%	15.5%	15.3%	0.12	−0.01
male ward 3	13.2%	12.0%	16.8%	12.9%	12.5%	0.14	−0.01
specialty wards	13.7%	16.7%	4.7%	13.8%	17.3%	−0.39	0.10
surgical wards	9.7%	10.4%	7.7%	9.7%	9.0%	−0.09	−0.02
CCI	5.47 (3.61)	5.54 (3.61)	5.24 (3.59)	5.48	5.27	−0.08	−0.06
Killip class							
1	56.2%	54.4%	61.7%	56.2%	56.0%	0.15	0.00
2	8.6%	8.2%	9.7%	8.7%	7.4%	0.05	−0.05
3	20.2%	20.1%	20.4%	19.8%	19.7%	0.01	0.00
4	15.0%	17.3%	8.3%	15.2%	16.8%	−0.27	0.04
Laboratory result							
Creatinine (μmol/L)	180.64 (210.16)	184.64 (214.01)	168.82 (192.07)	183.01	182.13	−0.08	0.00
Hemoglobin (g/L)	11.83 (2.47)	11.65(2.48)	12.36(2.38)	11.82	12.05	0.29	0.09
Platelet (1000/µL)	233.67 (95.57)	233.60(96.70)	233.89 (92.24)	233.39	234.83	0.00	0.02
RDW (%)	14.31 (1.91)	14.42 (2.00)	13.96 (1.55)	14.31	14.32	−0.26	0.00
High sensitivity troponin T (ng/L)	2156.22 (7086.72)	2347.12 (8036.76)	1576.82 (2693.75)	2117.57	1823.48	−0.13	−0.05
Bilirubin (μmol/L)	10.44 (9.06)	10.68 (9.70)	9.73 (6.81)	10.47	10.63	−0.11	0.02
eGFR (mL/min/1.73 m^2^)	57.50 (32.89)	56.72 (33.15)	60.04 (32.02)	57.27	56.81	0.10	−0.01
Neutrophil-lymphocyte ratio	7.60 (9.74)	8.02 (10.57)	6.34 (6.56)	7.62	7.74	−0.19	0.01

IPTW: inverse probability of treatment weighting. ACS: acute coronary syndrome. CAD: coronary artery disease. GI bleed: gastrointestinal bleeding. CKD: chronic kidney disease. COPD: chronic obstructive pulmonary disease. NSTEMI: non-ST elevation myocardial infarction. STEMI: ST-elevation myocardial infarction. PCI: percutaneous coronary intervention. CABG: coronary-artery bypass-grafting. RDW: red cell distribution width. eGFR: estimated glomerular filtration rate. CCI: Charlson Comorbidity Index. Diabetic microvascular ds: diabetic microvascular disease, which includes diabetic retinopathy, diabetic nephropathy and diabetic neuropathy.

**Table 2:** Difference in management in checklist and standard care after IPTW.

	Checklist	Standard	*p* Value
Treatment			
Aspirin	93.5%	80.4%	<0.0001
P2y_12_ inhibitor	88.1%	65.8%	<0.0001
P2y_12_ inhibitor type			
clopidogrel	68.7%	54.9%	0.0013
ticagrelor	19.4%	11.0%	0.003
ACEI/ARB/ARNI	81.4%	59.2%	<0.0001
Beta-blocker	75.0%	63.2%	0.0012
Statin	98.5%	83.9%	<0.0001
Statin intensity			
low intensity	9.5%	13.2%	0.1
moderate intensity	69.5%	52.3%	<0.0001
high intensity	19.5%	18.3%	0.68
Heparin	58.9%	85.0%	<0.0001
Early invasive	51.0%	20.7%	<0.0001
All subsequent revascularization	42.8%	28.2%	0.0001
* Estimated 90-day all-cause mortality	14.6%	25.1%	0.0006
Lost to FU	3.7%	2.0%	0.27

ACEI/ARB/ARNI: Angiotensin-converting enzyme inhibitor, angiotensin receptor blocker, or angiotensin receptor blocker-neprilysin inhibitor. Early invasive approach refers to cardiac catheterization within 72 h of index hospitalization while coronary revascularization includes subsequent revascularization scheduled as an elective admission after discharge. * While loss-to-follow-up may lead to unreported 90-day recurrent ACS and 90-day stroke, the estimated 90-day cumulative mortality rate remained valid as all-cause mortality data were retrieved directly from the territorial death registry, which included all reports of mortality in and outside of the healthcare setting.

**Table 3:** Sensitivity analysis.

Method	Adjusted Hazard Ratio	(95% CI)	Multivariate Cox Model *p*-Value	
All-factor multivariate cox regression model				
CCI tertile 1	0.83	0.53–1.29	0.399	
CCI tertile 2	0.62	0.41–0.94	0.025	
CCI tertile 3	0.72	0.50–1.03	0.068	
Overall	0.68	0.55–0.85	0.001	
IPTW with regression *				Adjusted log-rank test *p*-value
CCI tertile 1	0.70	0.41–1.18	0.183	0.34
CCI tertile 2	0.58	0.37–0.90	0.016	0.0552
CCI tertile 3	0.58	0.39–0.88	0.010	0.0074
Overall	0.64	0.49–0.84	0.001	0.0055
1:1 Propensity score matching				Log-rank test *p*-value
CCI tertile 1	0.83	0.50–1.36	0.452	0.4501
CCI tertile 2	0.63	0.41–0.99	0.044	0.0405
CCI tertile 3	0.75	0.50–1.15	0.187	0.1830
Overall	0.76	0.59–0.99	0.043	0.0416
CCI as continuous variable				
Checklist	0.61	0.46–0.81	0.001	
CCI (per point increase)	1.08	1.05–1.11	<0.001	

* For IPTW with regression, further adjustments were made by including covariates with SMD ≥ 0.1 after IPTW into a multivariate cox proportional hazard model for regression and adjustments of log-rank tests stratified for these covariates. The covariates adjusted for includes heart failure, presentation as unstable angina, prior ACS and admission to specialty ward units. CCI: Charlson Comorbidity Index.

## Data Availability

The data are not made publicly available owing to institutional policy restriction. Upon request, the data could be made available to researchers.

## Supplementary Materials

The following supporting information can be downloaded at: https://www.mdpi.com/article/10.3390/jcm14072469/s1, Figure S1: Kernal density plot of propensity score distribution before and after inverse probability of treatment weighting; Figure S2: Observed and predicted survival probability and Schoenfeld residuals for tests of proportional hazard assumption; Table S1: Distribution of propensity score in baseline population; Table S2: Test of proportional hazard assumption for individual variables. Graphical abstract: summary of study design, comorbidity in ACS patients, components of the implementation checklist, and results, including the use of GDMT and early invasive approach and 90-day events rates according to comorbidity burden and use of the checklist
