# Supplementary material for: Effectiveness of a Simplified Checklist to Overcome the Inertia of Treatment Implementation in ACS Patients with High Comorbidity Burden
Source: J Clin Med. 2025 Apr 4;14(7):2469. doi: 10.3390/jcm14072469 (PMC11990067; doi:10.3390/jcm14072469)
Supplement: Supplementary file 1 [file jcm-14-02469-s001.zip › jcm-3423675-supplementary.pdf]

Journal of
Clinical Medicine

MDPI

Supplemental Table S1. Distribution of propensity score in baseline population.

**Copyright:** © 2025 by the authors. Licensee MDPI, Basel, Switzerland. This article is an open access article distributed under the terms and conditions of the Creative Commons Attribution (CC BY) license (https://creativecommons.org/licenses/by/4.0/).	**standard**	**Checklist**	
Minimum value	0.0010149	0.0156889	Minimum value
P1	0.0052984	0.0303734	P1
P5	0.0174147	0.0875372	P5
P10	0.0295227	0.1384866	P10
P25	0.0666189	0.2447454	P25
P50	0.1568837	0.4093100	P50
P75	0.2862071	0.5754031	P75
P90	0.4281267	0.6866209	P90
P95	0.5583585	0.7236090	P95
P99	0.6895547	0.8050894	P99
Maximum value	0.7928281	0.9499483	Maximum value

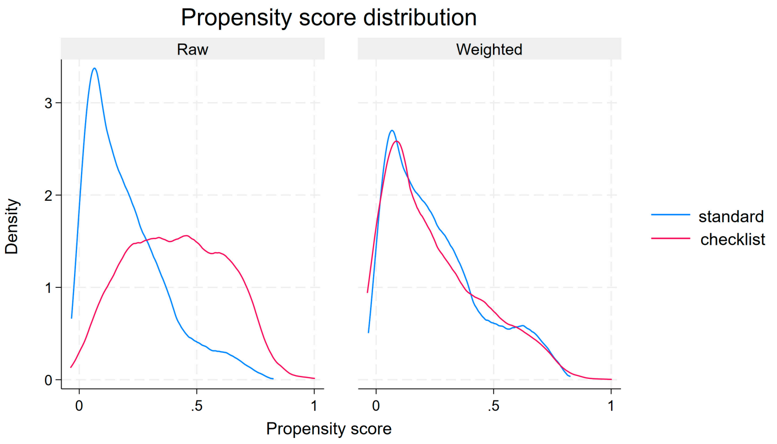

Supplemental Figure S1. Kernel density plot showing balancing of propensity score in checklist and standard care groups before (raw) and after(weighted) IPTW, showing good overlap of propensity scores after IPTW.

Logistic regression model for generating propensity score: covariates included: age, male sex, underlying malignancy, metastatic cancer, diabetes, microvascular ds diabetes, Familial hyperlipidemia, dementia, hypertension, prior acute coronary syndrome, prior coronary artery disease, Atrial fibrillation, heart failure, stroke, peripheral artery disease, prior GI bleeding, CKD stage: 1-5, Connective tissue ds, Syncope, COPD, Prior PCI, Prior CABG, ACS type:, NSTEMI, NSTEMI (secondary dx), unstable angina, Delayed presentation of STEMI, Admission year: 2016-2019, Admission unit: 6 internal medicine wards, specialty ward, surgical wards, CCI, Killip class: 1-4, Laboratory result, creatinine(µmol/L), hemoglobin (g/L) , platelet (1000/µL), Red cell distribution width (%), high sensitivity troponin T (ng/L), bilirubin (µmol/L), estimated glomerular filtration rate (eGFR) (mL/min/1.73 m2), Neutrophil-lymphocyte ratio.

Hosmer-Lemeshow goodness-of-fit test: p=0.5135

C-statistic (an area under the receiver operator curve): 0.7915. Pseudo R^2: 0.19

 https://doi.org/10.3390/jcm14072469

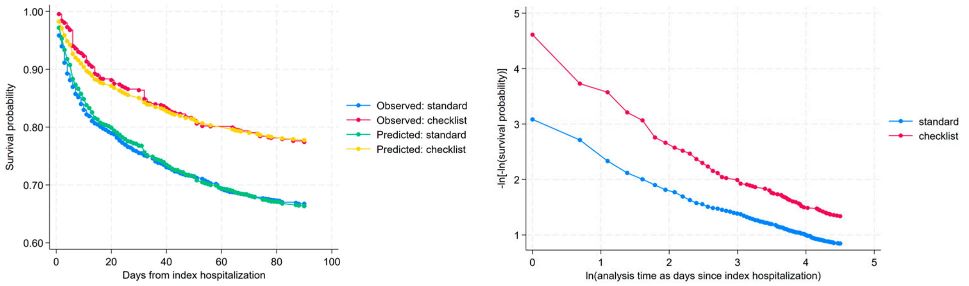

Supplemental Figure S2. left: plot of predicted and observed survival probability and right: plot of Schoenfeld residual over time, showing good overlap of observed and predicted survival probability as well as two parallel lines representing the Schoenfeld residues for checklist and standard groups. .

Supplemental table S2: test of proportional hazard assumption for individual variables. Time function: analysis time

**Variable**	**Dependency measure (ρ)**	**Likelihood ratio (chi-squared)**	**Degree of freedom**	**p-value (proportional hazard test)**
Checklist	0.07549	3.7	1	0.055
age	-0.04989	1.8	1	0.177
male sex	-0.00896	0.1	1	0.818
underlying malignancy	-0.00517	0	1	0.897
metastatic cancer	0.01819	0.2	1	0.646
diabetes	-0.01824	0.2	1	0.646
microvascular ds diabetes	0.04379	1.3	1	0.259
Familial hyperlipidemia	0.00822	0.1	1	0.831
dementia	0.01763	0.2	1	0.647
hypertension	0.04526	1.3	1	0.253
prior acute coronary syndrome	0.08047	4.2	1	0.04
prior coronary artery disease	0.02122	0.3	1	0.573
Atrial fibrillation	0.02831	0.5	1	0.461
heart failure	-0.04047	1.2	1	0.267
stroke	-0.00607	0	1	0.88
peripheral artery disease	-0.02251	0.3	1	0.563
prior GI bleeding	0.02104	0.3	1	0.586
CKD stage				
1	.	.	1	.
2	-0.02361	0.3	1	0.569
3	0.00075	0	1	0.986
4	0.0087	0	1	0.833
5	0.01728	0.2	1	0.672
Connective tissue ds	-0.00709	0	1	0.859
Syncope	0.06536	2.9	1	0.088
COPD	0.03439	0.8	1	0.37
Prior PCI	0.07595	3.8	1	0.051
Prior CABG	-0.06116	2.5	1	0.116
ACS type				
NSTEMI	.	.	1	.
NSTEMI (secondary dx)	0.05129	1.9	1	0.17
unstable angina	0.10042	5.8	1	0.016
Delayed presentation of STEMI	-0.04154	1.2	1	0.284
Admission year				
2016	.	.	1	.
2017	-0.06067	2.4	1	0.119
2018	-0.06002	2.4	1	0.125
2019	-0.0578	2.2	1	0.134
Admission unit				
female ward 1	.	.	1	.
female ward 2	0.02887	0.6	1	0.454
male ward 1	-0.00624	0	1	0.873
male ward 2	0.01347	0.1	1	0.732
female ward 3	0.01148	0.1	1	0.766
male ward 3	-0.01382	0.1	1	0.723
specialty wards	0.00056	0	1	0.989
surgical wards	0.01622	0.2	1	0.675
CCI	-0.01244	0.1	1	0.748
Killip class				
1	.	.	1	.
2	0.02443	0.4	1	0.528
3	0.04009	1.1	1	0.302
4	-0.0722	3.7	1	0.055
Laboratory result				
creatinine(μmol/L)	-0.02628	0.5	1	0.502
hemoglobin (g/L)	-0.04821	1.5	1	0.214
platelet (1000/μL)	-0.0102	0.1	1	0.784
Red cell distribution width (%)	0.01713	0.2	1	0.684

high sensitivity troponin T (ng/L)	0.02542	0.3	1	0.566
bilirubin (µmol/L)	-0.00793	0.1	1	0.812
eGFR (mL/min/1.73 m2)	0.00595	0	1	0.884
Neutrophil-lymphocyte ratio	-0.04237	1.3	1	0.263
Global test	62.72	51	0.1258	

The global test for proportional hazard test =0.1258 which is >0.05. For individual variables, p=0.055 for checklist >0.05, and p>0.05 for most variables except unstable angina as MI presentation (p=0.016) and prior ACS (0.04). Overall, the proportional hazard assumption is not violated.
